# Accuracy and reliability of the optoelectronic plethysmography and the heart rate systems for measuring breathing rates compared with the spirometer

**DOI:** 10.1038/s41598-022-23915-1

**Published:** 2022-11-10

**Authors:** Laurent Stubbe, Nicolas Houel, François Cottin

**Affiliations:** 1 Université Paris-Saclay, CIAMS EA 4532, 91405 Orsay, France; 2 Université d’Orléans, CIAMS EA 4532, 45067 Orléans, France; 3 ESO-Paris Recherche, Ecole Supérieure d’Ostéopathie – Paris, 77420 Champs Sur Marne, France; 4 Université de Reims Champagne-Ardenne, PSMS, Reims, France

**Keywords:** Chronic obstructive pulmonary disease, Biomedical engineering, Three-dimensional imaging

## Abstract

Measuring breathing rates without a mouthpiece is of interest in clinical settings. Electrocardiogram devices and, more recently, optoelectronic plethysmography (OEP) methods can estimate breathing rates with only a few electrodes or motion-capture markers placed on the patient. This study estimated and compared the accuracy and reliability of three non-invasive devices: an OEP system with 12 markers, an electrocardiogram device and the conventional spirometer. Using the three devices simultaneously, we recorded 72 six-minute epochs on supine subjects. Our results show that the OEP system has a very low limit of agreement and a bias lower than 0.4% compared with the spirometer, indicating that these devices can be used interchangeably. We observed comparable results for electrocardiogram devices. The OEP system facilitates breathing rate measurements and offers a more complete chest-lung volume analysis that can be easily associated with heart rate analysis without any synchronisation process, for useful features for clinical applications and intensive care.

## Introduction

Various studies have already focused on human physiological rates, including those involving metabolism, hormones, the autonomic nervous system and their interactions^1^. The estimation of human physiological rates is of interest for clinical research and health monitoring with regard to disease prevention^2^. The analysis of these rates help to better understand the interactions between human physiological systems from a macroscopic point of view^3–6^. In this context, measuring breathing rates without a mouthpiece is needed to monitor, treat and/or prevent various health conditions such as chronic obstructive pulmonary disease, post-thoracic surgery, ankylosis spondylitis, breathing kinematics in the context of spinal cord injuries^7,8^ and more recently for pulmonary complications in severe COVID-19 cases^9,10^.

Variation in various human biological rhythms, such as respiration, hearth rate, blood pressure, are currently estimated using non-invasive devices^11^. Spirometry is the standard method for monitoring breathing. For specific pathological care, monitoring the respiration rate without a mouthpiece has become mandatory. Over the past decade, methods based on electrocardiograms (ECGs) and plethysmograms have become accepted for estimate the breathing rate. For clinical monitoring, the global fast Fourier transform algorithm has been recognized for its accuracy and sensitivity in the identification of the main breathing and heart rates^11,12^. The usefulness of these two methods for monitoring breathing has been attributed to the relationship between thoracic motion and hearth rate, known as respiratory sinus arrhythmia^12^. Accordingly, optical fibre sensors are increasingly used to asses breathing rates because they can be associated with magnetic resonance imaging^13–15^. However, this technique has a relatively high limit of agreement (LOA) ranging from ± 0.45 to ± 2 breath/min (± 0.0075 to ± 0.0333 Hz)^13,14^. Similarly contact ultrasonic sensors monitor breathing in sleep apnoea syndromes^16,17^ or in conjunction with emotional state such as anger or happiness. This method has a Kappa coefficient of k = 0.38 compared with oronasal flow^17^. Infrared thermography can estimate normal breathing patterns^18–20^, because nasal and torso thermal signatures show a high cross-correlation (r = 0.98)^21^. Thermal signatures have an LOA of ± 0.5 s compared with inductance plethysmography^22^. Based on variation in chest wall velocity, a triaxial accelerometer indirectly measures breathing and hearth rate variability^23^, and these physiological signals are highly correlated (r = 0.96) with those estimated from chest deformation gauges and pulse oximeters^24^. However, accelerometers show time-cumulative errors compared with spirometer measurements^25^, and they have an LOA of ± 4 breath/min (± 0.0666 Hz) compared with ECGs^26^. Finally, although all these devices are more or less accurate in estimating human breathing rates, they do not provide information on the biomechanics associated with breathing rates.

Structured light plethysmography offers a better estimate of three-dimensional chest wall motion and its frequency^27,28^. This marker-less method can be used to reconstruct chest wall movements in clinical applications^29,30^. Respiratory volume monitored by structured light plethysmography correlates (R^2^ > 0.91) with spirometer measurements^27^. However to our knowledge, the accuracy and reliability of structured light plethysmography have not been investigated. Based on infrared cameras, optoelectronic plethysmography (OEP) is a motion-capture method that provides an accurate and reliable three-dimensional reconstruction of chest wall movements. The first OEP was developed 30 years ago^31,32^ based on video recordings of 32 passive motion-capture markers placed on the subject’s torso to measure three-dimensional chest volumes and the variability in chest wall surface motion and to estimate nine chest volumes. In these conditions, the 3D accuracy of the OEP system was SD = 0.06 mm^31^. Increasing the number of markers in the vertical and horizontal planes (some studies have used up to 89 markers) can improve accuracy^32,33^. However, due to its potential clinical applications, OEP was adapted with 24 markers and 9 virtual markers on the subject’s back to study breathing in newborns^34^. Recent studies have shown that increasing the number of markers always improved accuracy in OEP. According to Massaroni et al.^35^, bias and limit of agreement were lower when OEP is associated with 30 markers (bias = 0.056 l and LOA ± 0.35 l) compared with 89 markers (bias = 0.16 l and LOA ± 0.4 l). Moreover, OEP associated with 16 markers seems to be sufficient to monitor tidal volume in spontaneous breathing^36^, and other studies have shown that OEP associated with less than 16 markers can be used to estimate breathing rates and specific biomechanical parameters, such as the ratio between thoracic and abdominal breathing movements (13 markers)^37^ or sternal angle variation (6 markers).^38^. To our knowledge, only Shafiq and Veluvolu^39^ have used OEP associated with 16 markers to monitor breathing and cardiac frequencies, simultaneously according to the chest wall marker positions in Alnowan et al.^40^, with 12 of them presenting characteristic signals due to their proximal position on the diaphragm^39^. These 12 markers better predict diaphragm movements using abdominal chest volumes^41^ and provide estimates of breathing rates. Only two studies^34,42^ have compared the accuracy of OEP compared with the standard method on more than 10 subjects, (10 adults and 20 infants, respectively). Both studies used more than 16 retro-reflexives markers placed on the anterior torso (respectively 45 markers for adults and 24 markers for infants). To our knowledge, no study has assessed the accuracy and reliability of breathing rate estimates from a 12-marker OEP system compared with those from a standard method. Nowadays, Clinical use of OEP stay mainly limited to chest volume^33,38^. However, breathing frequency could be easily defined to prevent chest wall changes^43,44^ and dysfunctions^45,46^. Since 1990’s, OEP has a great interest in order to predict respiratory dysfunctions^45,46^, then to define changes of contribution in chest wall volume^33^.

The aim of the present study was to evaluate the accuracy and reliability of monitoring breathing rates using an OEP system and an ECG device compared with the standard spirometer. We tested whether controlled breathing rates estimated using OEP and ECG can reproduce spirometer measurements with the same accuracy and reliability.

## Methods

### Experimental design

Here, we estimated breathing rates from an OEP system as well as from ECG signals and heart rate (RR) intervals using an ECG device and compared them with spirometer measurements during audio-controlled subject breathing.

### Subjects

Twenty-nine volunteers participated in the present study. Subjects (17 females and 12 males; mean ± standard deviation (SD): age = 19.69 ± 1 years; height = 170.45 ± 9.59 cm; body mass = 61.79 ± 13.41 kg). The study was approved by the local ethics committee at the Ecole Superieure d’Osteopathie-Paris (France) and carried out in accordance with the Declaration of Helsinki (World Medical Declaration of Helsinki, 2013). All subjects were informed of the objectives of the study and signed the informed consent form before participating.

### Experimental setup

**Figure 1:**
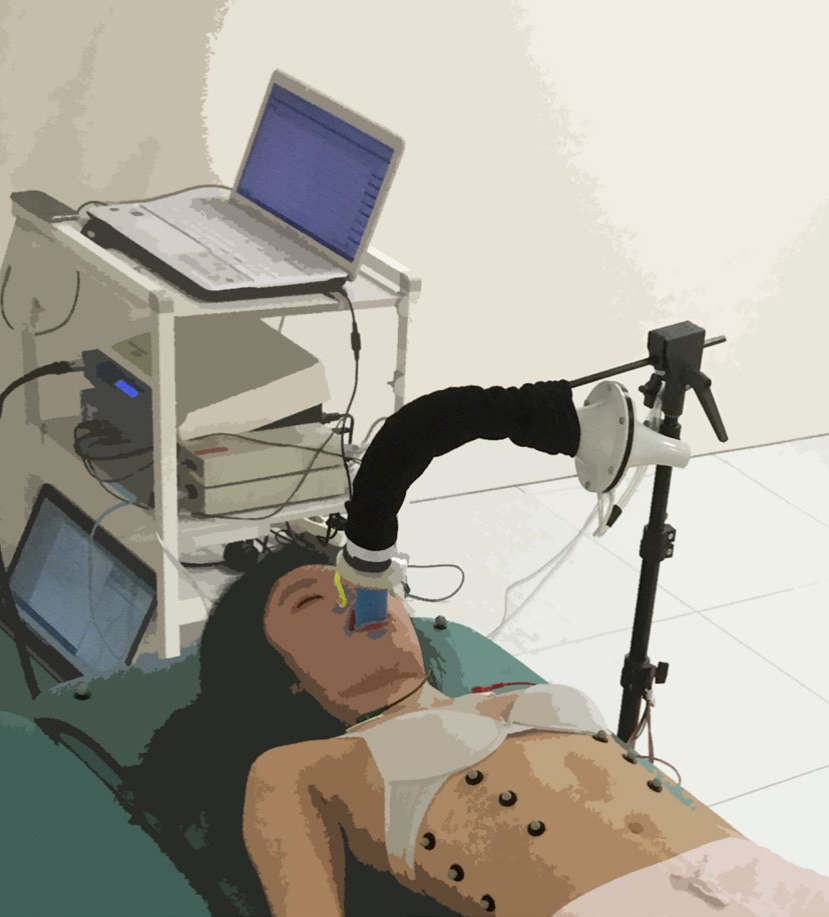
Subject in a supine position with spirometer mounted on a support to limit discomfort and stress.

The study took place in a well-ventilated, quiet room with constant temperature (= 22 °C), without ultraviolet disturbances. Subjects were instructed to refrain from smoking, and to avoid caffeine and other stimulants (medication or drugs) 72 h before the experiment^47^. Each subject was greeted and invited to relax without any stimulation in a resting room for 30 min according to the task force recommendations^2^. Then, the subject lay down in supine position on a large table to maximise relaxation and limit any stress associated with physiological monitoring. The subject was instructed to inhale and exhale at a frequency of 15 breath/min (0.25 Hz) in rhythm with an audio recording for 7 min followed by one breath at maximal tidal volume^47,48^. The subject performed the whole breathing exercise (audio-guided breathing and one maximal tidal volume breath) three times separated by a 7-min recovery period. During the whole breathing exercise, physiological parameters were recorded using an OEP system, an ECG and a spirometer. A spirometer was mounted on a hands-free support (Fig. 1) to limit discomfort and restraints (cf. Niérat et al.^49^).

### OEP procedure

**Figure 2:**
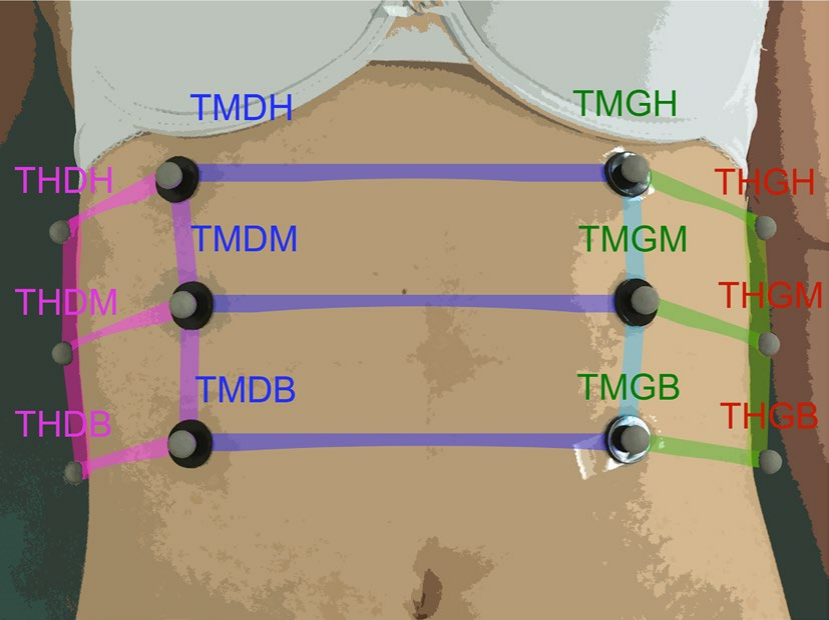
The 12 retro-reflexive marker positions on the subject at the intersections of six planes on the surface of the chest wall.

Twelve retro-reflexive markers (12 mm each) were placed on the chest (Fig. 2), in accordance with previous studies^31,39–41^. The 12 marker set were located around diaphragm zone that best characterizes all breathing possibilities in order to assess respiratory rate without influence of local contribution of chest wall volumes observed in pathological or normal subjects^33,41^. As example, thoracic and abdominal (Vab) contributions to total chest wall volumes significantly change from Vab = 43 ± 14% to Vab = 63 ± 14%, respectively for normal subjects and patients receiving pressure support ventilature^33^. Lateral asymmetric contributions to total chest wall volumes changes between 46 and 55% in patient with unilateral diaphragmatic weakness^50^.

**Figure 3:**
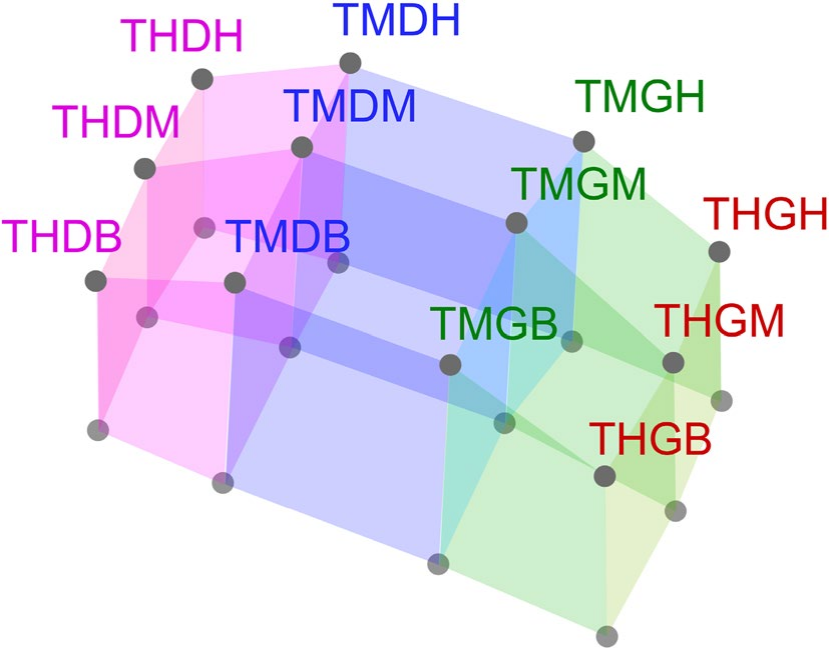
Plot of chest volume computed using six parallelepipeds. Each parallelepiped includes subject markers and its projection in the reference plane.

Markers were distributed on the surface of the chest wall at the 12 intersections of six planes: a median coronal plane crossing the chest, two sagittal medial-clavicular planes, a transverse subxiphoid plane, a transverse subcostal plane and a median transverse plane between the two previous transverse planes (see Fig. 3). For recording, three other markers were placed on the table to define a reference plane^33,34,42^.

Chest marker positions were recorded with eight infrared cameras (MXT10) at a 100 Hz sampling frequency. Cameras were time-synchronized using a Vicon Nexus 1.8.5 optoelectronic system and with a MX Giganet link^51^. Standard deviation of the reference plane^31^ was equal to 0.062 mm.

### Spirometer and ECG procedures

During each session, breathing was continuously recorded using a spirometer. Cardiovascular signals (ECG) were also continuously recorded at 1000 Hz. RR-interval time series were extracted from raw ECG signals^52^. Respiration and cardiovascular signals were recorded, digitalized and synchronized using a Power-Lab 8/35 device (Human respiratory kit with spirometer and ECG Bio Amps, ADInstrument^®^). Calibrations were performed according to the manufacturer’s instructions before each test on each subject.

### Synchronization setup

Kinematics, ECG and spirometer data were time-synchronized by determining the minimum local signal during the exhale at tidal maximal volume, similar to the procedure in Lo Presti et al.^53^. For each signal, a 6-min epoch before the synchronization point was selected. A fast Fourier transform was performed on each signal included in the 6-min epoch.

### OEP processing

**Figure 4:**
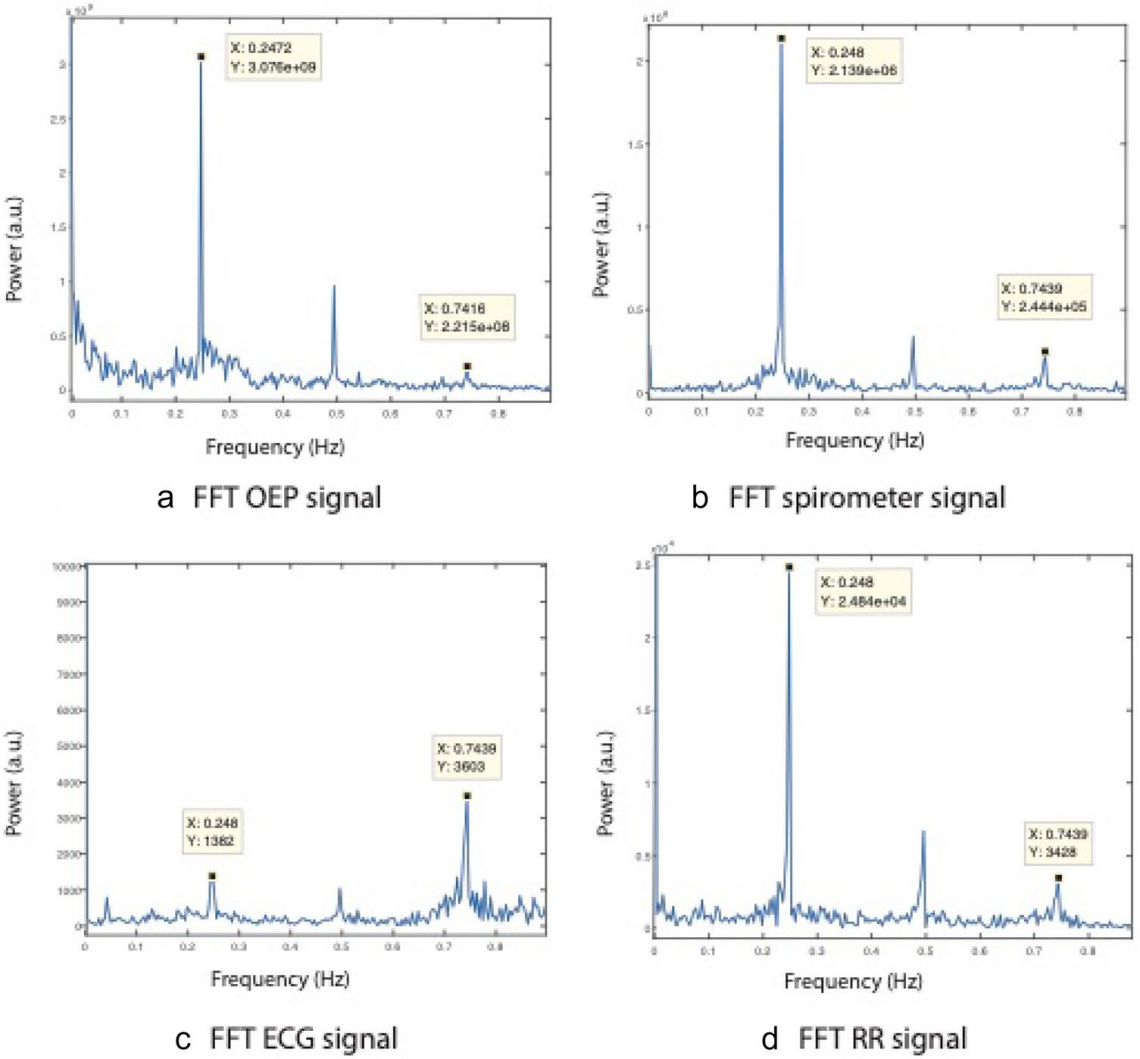
Plots of subjects’ breathing frequencies nearest to 0.25 Hz (15 breath/min) during the controlled 6-min breathing exercise performed using a fast Fourier transform. (**a**) OEP signal (frequency = 0.2472 Hz or 14.83 breath/min); (**b**) spirometer data (frequency = 0.248 Hz or 14.88 breath/min); (**c**) ECG data (frequency = 0.248 Hz or 14.88 breath/min) and (**d**) with RR-interval time series (frequency = 0.248 Hz or 14.88 breath/min).

Chest volume was first computed using the 12 subject markers and the three reference plane markers. The projection of the position of the 12 markers was calculated in the reference plane. Six volumes were computed using the parallelepiped equation (Fig. 3). Chest volume was equal to the sum of the six previous parallelepipeds. Fast Fourier transform was performed on the chest volume signal for each 6-min epoch. The local maximum amplitude frequency signal nearest to 15 breath/min (0.25 Hz) was extracted (Fig. 4a). This frequency matches the subject’s own breathing rate during the controlled breathing exercise.

### Physiological data processing

For each physiological data point (spirometer, ECG and RR series), the fast Fourier transform was applied to the same 6-min epoch used for OEP recordings. The same local maximal amplitude frequency signal nearest to 15 breath/min (0.25 Hz) was extracted (Fig. 4b–d). All data processing was carried out using MATLAB^®^ 2018.

### Statistical analysis

The mean and standard deviation of breathing rates were computed. Reliability and agreement between OEP and ECG estimations were compared with spirometer data according to Kottner et al.^54^ recommendations. Correlation coefficients (r) were used to estimate the relationship between breathing rate data from OEP, ECG and RR with spirometer measurements. Testing the mean against a constant reference value was done to compare OEP as well as ECG and RR with spirometer values. The threshold alpha value was set to 0.05. A Bland–Altman plot was used to define accuracy and reliability between the OEP- ECG- or RR-based and spirometer breathing rates^55,56^.

## Results

In all, 81 recordings were taken with the three devices (OEP, ECG (ECG and RR signals), spirometer). Nine records were excluded from the OEP data due to insufficient or absence of maximal tidal volume, thereby limiting the synchronization process. Ultimately, 72 simultaneous recordings were analysed based on OEP signals and spirometer measurements and 81 simultaneous recordings were analysed based on ECG signals and spirometer measurements.

### OEP versus spirometer analysis

**Figure 5:**
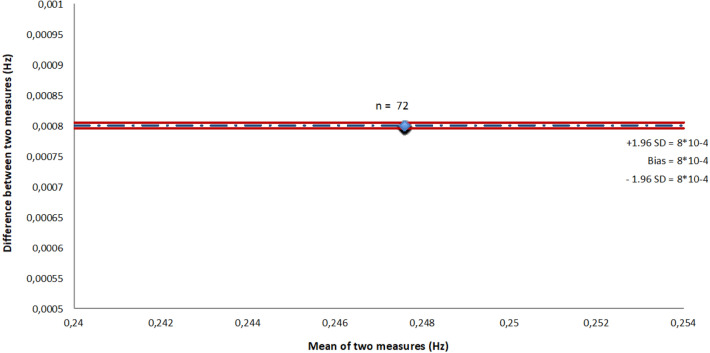
Plot of Bland–Altman showing the bias (broken line) and 95% limits of agreement (continuous line) between OEP chest volume frequencies and spirometer breathing rates.

The mean (± SD) OEP and spirometer breathing rates were respectively equal to 0.2472 ± 0 Hz (14.832 ± 0 breath/min) and 0.248 ± 0 Hz (14.832 ± 0 breath/min). Correlation coefficients showed a high values (r = 1, p < 0.001). The Bland–Altman plot indicated a significant bias equal to 8 × 10^–4^ Hz (0.05 breath/min) and an LOA of ± 0 Hz (0 breath/min) (Fig. 5). Figure 4a,b showed that breathing rates estimated using OEP signals were as strong as those based on spirometer signals due to the high amplitude value in the frequency domain.

### ECG versus spirometer analysis

**Figure 6:**
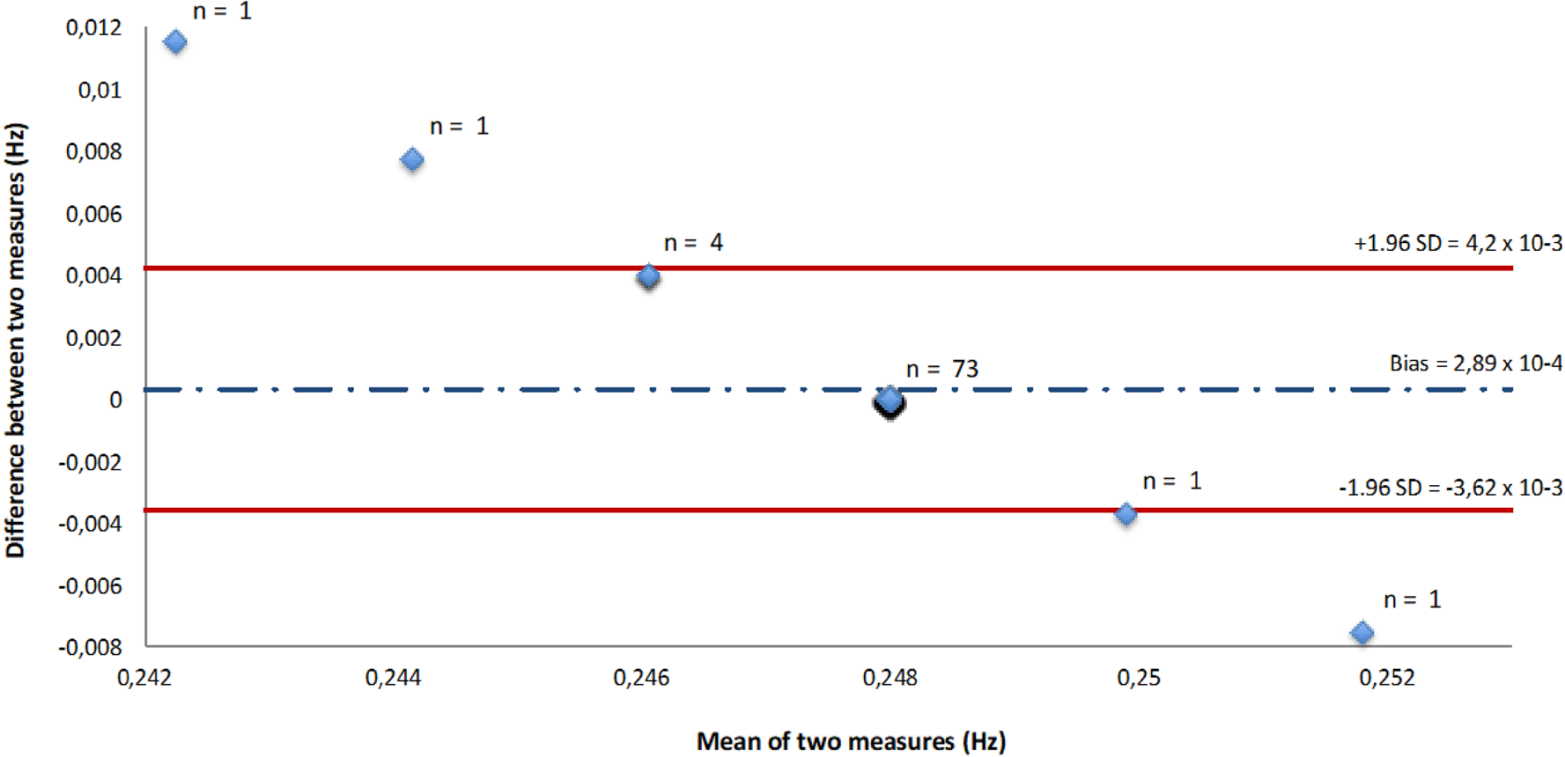
Plot of Bland–Altman showing bias (broken line) and 95% limits of agreement (continuous line) on breathing frequency values between ECG and spirometer.

The mean (± SD) ECG and spirometer breathing rates were respectively equal to 0.2477 ± 0.0019 Hz (14.862 ± 0.11 breath/min) and 0.248 ± 0 Hz (14.88 ± 0 breath/min). Testing the mean against the constant reference value showed no significant differences between ECG and spirometer data (p = 0.195). Correlation coefficients were high (r = 1, p < 0.001). A Bland–Altman plot showed a significative bias equal to 2.89 × 10^–4^ Hz (0.02 breath/min) and an LOA of ± 3.91 × 10^–3^ Hz (± 0.23 breath/min) (Fig. 6). Figures 4b,c show that breathing to hearth rates ratio estimated from ECG signal had a lower value than the breathing to hearth rates ratio given by the spirometer in the frequency domain.

### RR versus spirometer analysis

**Figure 7:**
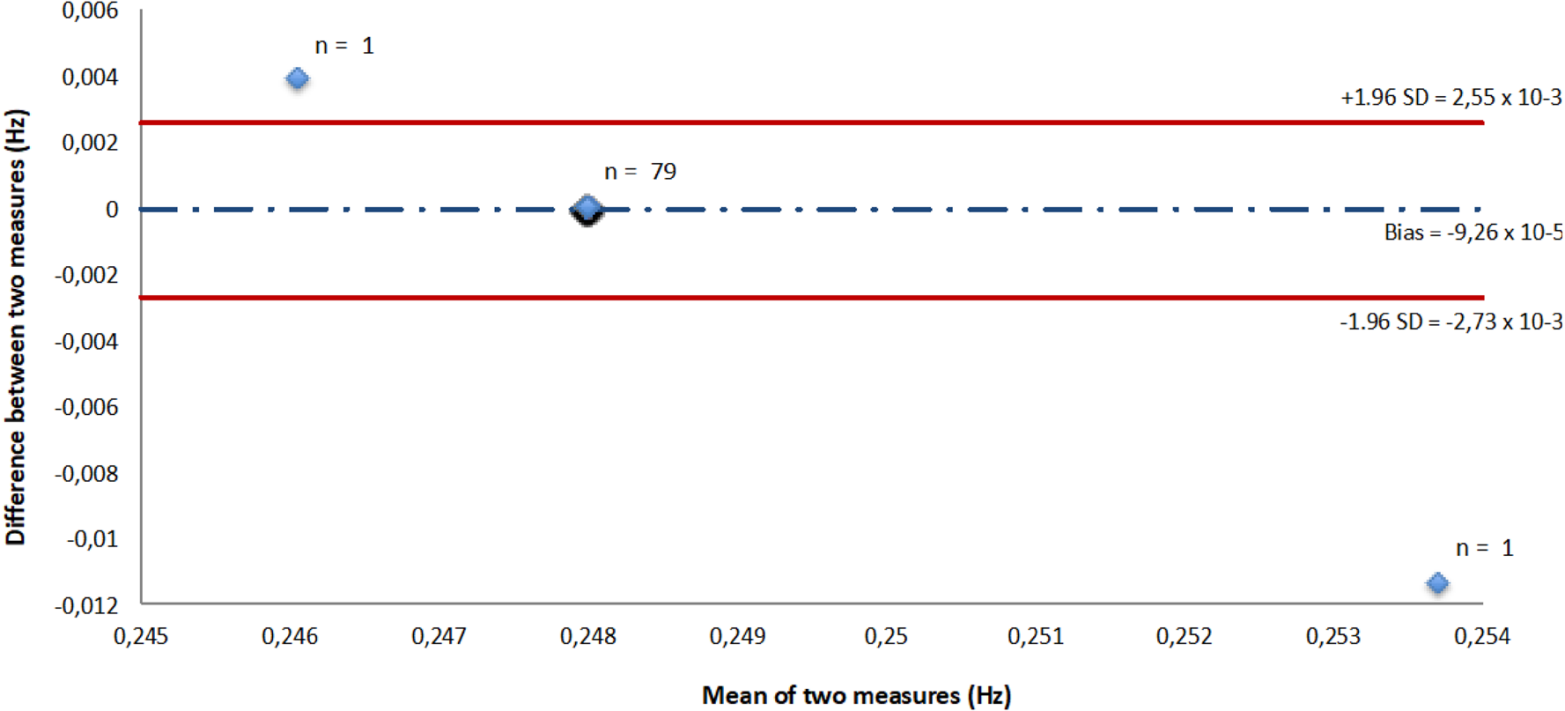
Plot of Bland–Altman showing bias (broken line) and 95% limits of agreement (continuous line) on breathing frequency values between RR and spirometer.

The mean (± SD) RR and spirometer breathing rates were respectively equal to 0.248 ± 0.0013 Hz (14.88 ± 0.08 breath/min) and 0.248 ± 0 Hz (14.88 ± 0 breath/min). Testing the mean against the constant reference value showed no significant difference between RR and spirometer data (p = 0.537). The correlation coefficient was high (r = 1, p < 0.001). The Bland–Altman plot showed a significant bias equal to − 9.26 × 10^–5^ Hz (− 0.005 breath/min) and an LOA of ± 2.64 × 10^–3^ Hz (0.16 breath/min) (Fig. 7). Figures 4b,d show that breathing to hearth rates ratio estimated from the RR signal was as strong as breathing to hearth rates ratio from the spirometer in the frequency domain.

## Discussion

The aim of the present study was to estimate the accuracy and reliability of breathing rates estimated using an OEP system or an ECG compared with those measured using the standard spirometer method. The results of the present study showed that breathing rates estimated from an OEP system based on only 12 motion-capture markers or from ECG closely corroborate spirometer-based measurements.

### Agreement between OEP analysis and spirometer measurements

Breathing rates estimated using an OEP system were in agreement with spirometer recordings taken following Kottner et al.^54^ recommendations, with an LOA of ± 0 Hz (± 0 breath/min) and a bias of 8 × 10^–4^ Hz (0.05 breath/min). This bias represents 0.32% of the reference value. Previous studies have already shown the accuracy and reliability of kinematics analysis for estimating chest volume^31,32,34^. However, to our knowledge, the results of previous studies were based on 45 markers and measurements on fewer than 10 adults. The improved of OEP estimates of breathing rates can be attributed to our calibration process that provides a space-volume reconstruction with a accuracy of ± 0.062 mm. This level of accuracy has been already observed in other studies on chest volume^31^ and spinal curve^51^. The main advantage of OEP analysis is to provide non-invasive biomedical estimates of chest-wall compartments and therefore variations in lung volume^7,8^, particularly for mechanically ventilated patients^33^, monitoring breathing in preterm and term infants^57^, or pulmonary complications in severe COVID-19 cases^9,10^. In particular, OEP analysis provides simultaneous estimates of breathing and heart rates without a synchronization process and without the stress associated with the use of a mouthpiece, as usually observed with the spirometer device^49,53^.

### Agreement between ECG (ECG, RR interval) and spirometer recordings

Breathing rates have already been estimated using ECG (ECG signals and RR intervals) recordings^12,58^. Breathing rates estimated from the ECG signal were in agreement with spirometer measurements with an LOA of ± 3.91 × 10^–3^ Hz (0.23 breath/min) and a bias equal to 2.89 × 10^–4^ Hz (0.02 breath/min). The correlation between both devices was very high. Breathing rates estimated from an RR-interval analysis is in agreement with spirometer measurements with an LOA of ± 2.64 × 10^–3^ Hz (± 0.16 breath/min) and a bias equal to − 9.26 × 10^–5^ Hz (− 0.005 breath/min). Biases for ECG and RR were respectively 0.12% and 0.04% of the reference value. The correlation of the values from both devices was also very high. No significant differences were observed between ECG signals (ECG, RR intervals) and spirometer data. These results are in agreement with previous studies that showed that breathing rate interacts with heart rate and the RR interval^12,59,60^. Moreover, breathing rates are usually recorded during heart rate monitoring sessions that use ECGs (ECG signals, RR intervals)^58,59^. Our results indicate that breathing rates estimated using an RR analysis show less bias and a better LOA than ECG analysis. Moreover, Fig. 4 demonstrates that breathing rates were more easily detected in the frequency domain using RR-interval analysis. These results confirm the advantage of performing RR analysis to better monitor and diagnose heart rate and breathing variability compared with the usual clinical analysis based on the time and the frequency domains^11,58^.

Both OEP- and ECG-based analyses show accuracy and reliability on par with the spirometer. Due to its negligible LOA and its bias lower than 0.4%, OEP analysis offers more opportunities for biomedical monitoring than ECG devices. OEP signals provide higher power of detection of breathing frequency and more opportunities to define chest lung volume and the breathing rate.

The main limitation in the present study was the use of audio recordings to guide the subject’s breathing. Audio recordings has been already used in previous studies to better estimate both breathing and heart rate^59–61^. Here audio recordings were to define breathing and heart rate rhythms more easily without complex algorithms other than the fast Fourier transform. If respiratory frequency changes are associated with various internal systems interactions and external stressors and illness^46^, the choice to use a controlled 0.25 Hz (15 breath/min) frequency in the present study was motivated by assessing the signal to noise ratio that was the most representative of the respiratory signal assessment^2,46^. Various studies have shown that controlled breathing rate at 0.25 Hz (15 breath/min) was the most representative of spontaneous breathing in normal subject^46,48,62^. The controlled breathing frequency experimental design at 0.25 Hz has been already used in order to assess breathing in various conditions^47,62,63^. According to Nicolo et al.^46^, OEP and ECG remain the best methods that could monitor accurately breathing rate. On a metrological point of view, estimating respiratory rate in controlled conditions with the best signal to noise ratio is needed before computing different algorithms based on auto-correlations or wavelets^46^. However, if in the present study, measurements have been made under controlled breathing rate (15 breath/min) that is advantageous for periodic signal analysis, further complementary studies will be realized in order to explore various breathing patterns observed in clinical care. That breathing patterns will be observed under spontaneous breathing, irregular over breathing, breathings with fluctuating tidal volume and frequency including apnea. Moreover, complementary signal processing will be used to improve the estimates of breathing frequency and heart rate variability according to the clinical purposes or to define consensus conditions^2^.

## Conclusion

The present study showed that OEP, and ECG devices can be used interchangeably with respect to the standard spirometer when breathing frequency monitoring is required. All devices showed very low LOA values and bias lower than 1% compared with the standard spirometer method. The OEP system offers many possibilities to estimate biomedical signals simultaneously (e.g. breathing and heart rates). Similarly to ECG and RR-interval analysis, OEP opens the way for new monitoring solutions associated with specific clinical applications. The non-invasiveness of the kinematics device (only passive markers are placed on the subject’s chest) is particularly attractive feature for intensive care and preterm infant care.
